# A paradoxical association of an oxytocin receptor gene polymorphism: early-life adversity and vulnerability to depression

**DOI:** 10.3389/fnins.2013.00128

**Published:** 2013-07-23

**Authors:** Robyn J. McQuaid, Opal A. McInnis, John D. Stead, Kimberly Matheson, Hymie Anisman

**Affiliations:** ^1^Department of Neuroscience, Carleton UniversityOttawa, ON, Canada; ^2^Department of Psychology, Carleton UniversityOttawa, ON, Canada

**Keywords:** depression, distrust, early-life, maltreatment, oxytocin, SNP, stress

## Abstract

Several prosocial behaviors may be influenced by the hormone oxytocin. In line with this perspective, the oxytocin receptor (OXTR) gene single nucleotide polymorphism (SNP), rs53576, has been associated with a broad range of social behaviors. In this regard, the G allele of the OXTR SNP has been accompanied by beneficial attributes such as increased empathy, optimism, and trust. In the current study among university students (*N* = 288), it was shown that early-life maltreatment was associated with depressive symptoms, and that the OXTR genotype moderated this relationship, such that under high levels of childhood maltreatment, only individuals with GG/GA genotype demonstrated increased depressive symptomatology compared to those with the AA genotype. In addition, the role of distrust in mediating the relation between childhood maltreatment and depression seemed to be more important among G allele carriers compared to individuals with the AA genotype. Thus, a breach in trust (i.e., in the case of early-life abuse or neglect) may have a more deleterious effect among G carriers, who have been characterized as more prosocial and attuned to social cues. The data suggested that G carriers of the OXTR might favor social sensitivity and thus might have been more vulnerable to the effects of early-life adversity.

## Introduction

Oxytocin, a neuropeptide produced in the hypothalamus, is involved in a broad range of physiological and behavioral processes. The role of oxytocin in pair-bonding and reproductive behaviors has been well-established (Carter, 1998; Gimpl and Fahrenholz, 2001), and this peptidergic system has become the focus of understanding the biological processes underlying prosocial behaviors (Donaldson and Young, 2008). In this regard, intranasal oxytocin has been shown to increase trusting behavior (Kosfeld et al., 2005), positive communication (Ditzen et al., 2009), and in-group favoritism (De Dreu et al., 2011). Furthermore, intranasal oxytocin has been associated with reduced social stress reactivity (Heinrichs et al., 2003), and attenuated amygdala activity in response to emotional stimuli (Domes et al., 2007).

In addition to intranasal manipulations of oxytocin, studies examining human social behaviors have also recently focused on the oxytocin receptor gene (OXTR) located on chromosome 3p25 (Inoue et al., 1994). A single nucleotide polymorphism (SNP), rs53576, involving a guanine (G) to adenine (A) substitution located in the third intron of the OXTR has emerged as an important candidate in the understanding of human social behaviors. Individuals homozygous for the G allele compared to those with the GA or AA genotypes exhibit greater empathy and an increased ability to detect emotion from pictures of human faces in which only the eyes were shown (Rodrigues et al., 2009), greater maternal sensitivity (Bakermans-Kranenburg and van Ijzendoorn, 2008) and higher self-esteem and optimism (Saphire-Bernstein et al., 2011). However, optimism was not associated with the OXTR rs53576 in a large cohort of Caucasian women (Cornelis et al., 2012). It was also revealed that in a sample of Caucasian males, those with the GG genotype displayed higher trust-related behaviors in comparison to A allele carriers (Krueger et al., 2012). Individuals with one or two copies of the G allele also showed lower cortisol levels compared to individuals with the AA genotype assessed in the Trier Social Stress Test after receiving social support (Chen et al., 2011). Furthermore, the GG/GA genotypes seek more emotional social support in Caucasian but not Asian participants (Kim et al., 2010) and have higher positive affect (Lucht et al., 2009) compared to AA genotypes.

Together, these findings indicate that having one or two copies of the G allele is associated with several positive features. However, the possibility exists that the seemingly positive effects of being a G carrier might be absent under conditions of adversity, especially those that involve negative early-life experiences. For instance, in an African American sample GG carriers exposed to severe childhood maltreatment displayed greater disorganized attachments styles and increased risk for emotional dysregulation compared to GA/AA carriers (Bradley et al., 2011). As well, among a Caucasian sample of depressed individuals, the GG genotype was associated with higher levels of adult separation anxiety compared to A carriers (Costa et al., 2009). It seems that in the face of adversity, the more prosocial and socially attuned individuals may be more sensitive and therefore more likely to be affected by adverse experiences such as abuse.

It might be expected that although the G allele has been associated with greater trust, a violation of that trust, as in the case of early-life abuse and neglect could be more upsetting for those individuals. Accordingly, in the present study we examined the OXTR SNP in relation to childhood maltreatment and mental health outcomes in a culturally diverse sample. It was predicted that individuals with one or two copies of the G allele who self-reported early-life maltreatment would display elevated depression scores compared to those with two copies of the A allele. Furthermore, the betrayal associated with childhood maltreatment may culminate into a general distrust for others (Doyle, 2001) which may have important ramifications related to depression (Lynch and Cicchetti, 1998). Thus, we predicted that levels of distrust may be a mediator through which maltreatment might lead to depressive symptoms. Further, given that OXTR has been implicated in trust-related behaviors (Krueger et al., 2012), we hypothesized that feelings of distrust following maltreatment would be more detrimental for those with a G allele.

## Methods

### Participants

Participants included 288 Carleton University first and second year students (213 females and 75 males), with a mean age of 19.99 (*SD* = 3.17) who were recruited through the university's online computerized recruitment system. Self-reported ethnicity included White (58.0%, *n* = 167), Black (11.8%, *n* = 34), Asian (8%, *n* = 23), Arab (5.7%, *n* = 17), South Asian (5.6%, *n* = 16), South East Asian (2.1%, *n* = 6), Latin American (1.4%, *n* = 4), Aboriginal (1.4%, *n* = 4), and other (e.g., mixed ethnicity, 5.9%, *n* = 17).

### Procedure

Once signed informed consent was obtained, participants responded to a series of demographic questions as well as measures of current depressive symptoms, childhood maltreatment, and distrust and cynicism. Saliva samples for DNA genotyping were taken following the questionnaires. Upon completion of the study, which took up to 1 h to complete, participants were debriefed and compensated with course credit. All procedures in the current study were approved by the Carleton University Ethics Committee for Psychological Research.

### Genotyping

Samples for genotyping were collected using Oragene OG-500 collection kits (DNA Genotek, Inc., Ottawa, ON, Canada). Genomic DNA was extracted from the sample collection kit according to the manufacturer's instructions and diluted to approximately equal concentration (20 ng/μL). Genotyping was conducted using quantitative polymerase chain reaction (qPCR). Amplification reactions were performed in a total volume of 15 μ l, containing ~1 μ L (20 ng) of genomic template, 0.6 μ L of each primer (concentration 10 μ M), 1.2 μ L of dNTP, 1.5 μ L 10X Buffer, 1.5 μ L of MgCl_2_, 0.3 μ L of Salmon Sperm DNA, 0.15 μ L of Taq polymerase, 0.015 of SYBR green, and 8.135 μ L of water. All q-PCR plates were run in duplicate. Following this, all qPCR products were electrophoresed on 2% agarose gel and then visualized to verify qPCR results. The Bio-Rad Iq5 Primer sequences used for qPCR were as follows: OXTR F1 forward: TCCCTGTTTCTGTGGGACTGAGGAC, OXTR F2 forward: TCCCTGTTTCTGTGGGACTGAGGAT, OXTR reverse: ACCCAAGAGGCTGGTTTGGGGTT.

The allele distribution of the OXTR polymorphism was 118 GG individuals (22 male, 96 female) and 119 GA (38 male, 81 female) and 43 AA individuals (14 male, 29 female). The genotype distributions met Hardy-Weinberg Equilibrium expectations, χ^2^_(1)_ = 1.99, *p* = 0.16. Based on earlier studies, there was reason to collapse across the GG and GA genotypes, but there was also precedent for collapsing across the GA and AA carriers. In the present study the depressive scores associated with the A allele were somewhat lower than among G carriers (AA compared to G carriers), albeit not significantly so, and thus the AA recessive allele was considered in comparison to the pooled G carriers. As will be seen, this approach fit the data better than the alternative procedure, which did not distinguish between these conditions on any of the variables measured in the present investigation. This same procedure was used in studies showing that individuals with the GG/GA genotypes seek more emotional social support (Kim et al., 2010), have higher positive affect (Lucht et al., 2009), and showed lower cortisol responses to stress after social support compared to individuals with two copies of the A allele (Chen et al., 2011).

Eight individuals were excluded from analyses including genotype because we were unable to determine a genotype from the samples provided. No significant differences were found between the genotype groups based on sex, χ^2^_(1)_ = 0.98, *p* = 0.32, and past or current mental disorders, χ^2^_(1)_ = 0.004, *p* = 0.95. A trend was apparent for differences between genotypes based on self-reported ethnicity, χ^2^_(8)_ = 14.13, *p* = 0.08. This is not surprising, as the distribution of the OXTR polymorphism differs between ethnicities. More specifically, among Asian ethnic groups the AA genotype is most prevalent, whereas it is least prevalent among Caucasians (Kim et al., 2011; Saphire-Bernstein et al., 2011). Similarly, in the current study, of the 21 Asian participants whose samples could be genotyped, eight of these were of the AA genotype, which is a much higher frequency than that found among Caucasians. Thus, as the Asian OXTR genotype distributions differed from that of Caucasian individuals, all of the main analyses were performed both with and without Asian participants. In both instances the observed results were very similar.

### Measures

#### Depressive symptoms

The 21-item Beck Depression inventory (BDI) (Beck et al., 1961) was used to assess depressive symptoms. For each item participants responded to one of four options which ranged from low to high depression symptomatology. Total scores were calculated by summing across all items (α = 0.91).

#### Childhood maltreatment

The 31-item Childhood Maltreatment Questionnaire (short form) (Demare, 1996) assessed levels of maltreatment comprising psychological (α = 0.95), physical (α = 0.90), and sexual abuse (α = 0.89) as well as neglect (α = 0.85). Each item can be rated from 1 (never) to 5 (very often) indicating the frequency of experiences.

#### Distrust and cynicism

The 8-item Distrust and Cynicism Scale (derived from the Cook–Medley Hostility Scale) has been used as a reliable and valid measure of cynical distrust (Greenglass and Julkunen, 1989, 1991). Each item can be rated from 0 (completely disagree) to 3 (completely agree). Total scores were calculated by taking the mean across all items (α = 0.82). Items such as; *“It is safer to trust nobody”* can be found in this scale.

## Statistical analyses

The statistical analyses were performed using SPSS for Windows 18.0 (SPSS Science, Chicago, IL, USA). Statistical significance was determined at *p* < 0.05 (two-tailed). Analyses assessing differences on depression scores, childhood maltreatment, and distrust were assessed using independent samples *t*-tests. Correlational analysis was performed using Pearson product moment correlations. Moderations were analyzed using hierarchical linear regressions, and the significant moderations were followed up using a web utility for simple slopes (Preacher et al., 2006). Mediation analyses were conducted using Sobel's test for estimating indirect effects (Preacher and Hayes, 2004). Moderated mediation analyses were conducted using bootstrapping procedures and confidence intervals based on 5000 resamples (Preacher et al., 2007). In all regression analyses standardized scores were used.

## Results

The depression scores among individuals with the GG and AG genotype were very similar to one another (*M* = 9.66; *SE* = 0.75 and *M* = 9.15; *SE* = 0.74, respectively) and although somewhat elevated compared to that of the AA genotype (*M* = 7.58; *SE* = 1.23), these groups did not significantly differ from one another, *t*_(1, 278)_ = 1.36, *p* = 0.18. Childhood maltreatment scores also did not differ based on genotype for total maltreatment scores, *t*_(1, 51.3)_ = −0.77, *p* = 0.45, or on any subscales of maltreatment, including physical abuse, *t*_(1, 51.3)_ = −0.70, *p* = 0.49, psychological abuse, *t*_(1, 278)_ = −0.67, *p* = 0.50, sexual abuse, *t*_(1, 42.4)_ = −0.93, *p* = 0.36, and neglect, *t*_(1, 50.1)_ = −0.96, *p* = 0.34. There were too few participants who reported sexual abuse (*N* = 2) to provide meaningful results. As seen in Table 1, levels of maltreatment experienced in the current study were appreciable, with the most common form of abuse being of a psychological nature, whereas neglect and physical abuse were less common. In the present study ~10% of participants reported experiencing physical abuse or neglect at least sometimes and up to very often. Additionally, up to 30% reported experiences of psychological abuse to the same degree. Furthermore, there were no differences on levels of distrust, *t*_(1, 278)_ = −0.27, *p* = 0.79, between genotype groups. For the Student *t*-test, the *p*-value for equal variances not assumed was reported when Levene's test was significant (*p* < 0.05).

**Table 1:** **Percentage of childhood maltreatment experienced**.

**Childhood maltreatment**	**Never/Rarely (%)**	**Sometimes (%)**	**Often/Very often (%)**
Psychological	70.7	15.0	14.3
Neglect	90.2	4.9	4.9
Physical	89.2	7.1	3.7

Females were found to have higher depressive scores than males, *t*_(1, 163.6)_ = 2.60, *p* = 0.01, and they also reported higher levels of childhood maltreatment, *t*_(1, 225.4)_ = 2.49, *p* = 0.01. However, there were no gender differences on distrust levels *t*_(1, 286)_ = −0.15, *p* = 0.89, nor was the Gender × Gene interaction significant in relation to depressive symptoms, childhood maltreatment, or distrust. Furthermore, in an effort to control for the potential influence of population stratification, analyses assessing Ethnicity × Gene interactions on depressive symptoms, distrust, and childhood maltreatment were not found to be significant. In this regard, these analyses included all nine ethnicities as one of the factors, as well as a further analysis in which these ethnicities were collapsed into 5 ethnic conditions.

The relation between childhood maltreatment and depressive symptoms revealed that total scores on childhood maltreatment were related to severity of depressive symptoms *r* = 0.44, *p* < 0.001. Likewise, psychological abuse, *r* = 0.46, *p* < 0.001, physical abuse, *r* = 0.30, *p* < 0.001, and neglect, *r* = 0.34, *p* < 0.001, were positively related to depression scores. The possible moderating effect of genotype was explored regarding the relationship between total childhood maltreatment scores, psychological abuse, physical abuse, and neglect with symptoms of depression.

**Figure 1:**
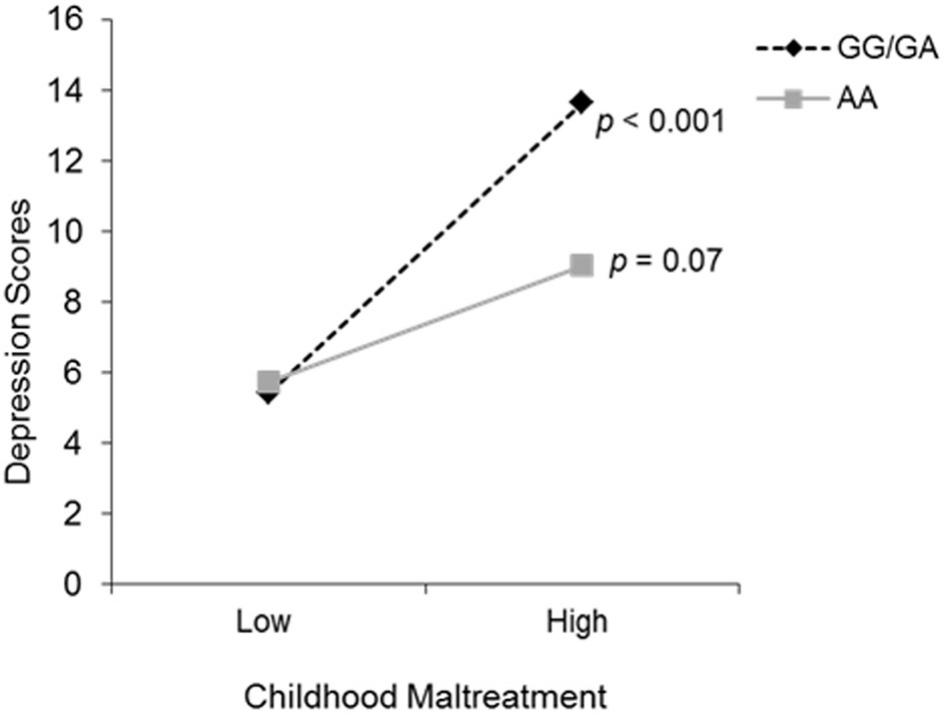
**The relation between childhood maltreatment and depression scores as a function of the OXTR rs53576 genotype (GG/GA vs. AA)**. The simple slopes analyses revealed that genotype groups did not differ at lower levels of childhood maltreatment. However, depressive symptoms increased significantly when higher levels of childhood maltreatment were experienced, but only among those individuals with the GG/GA genotype.

To examine the possible interaction between total childhood maltreatment scores and depressive symptoms with OXTR genotype, a hierarchical linear regression was conducted. Genotype and total maltreatment scores were entered on the first step, and the Genotype × maltreatment interaction term was entered on the second step. The moderating role of OXTR genotype on the relation between childhood maltreatment and depression was significant, Δ R^2^ = 0.02, *b* = −2.47, *t* = −2.42, *p* = 0.02. Follow up simple slope analyses (Preacher et al., 2006), as shown in Figure 1, revealed that levels of depressive symptoms did not differ between genotypes at low levels of childhood maltreatment. However, at high levels of childhood maltreatment, depressive symptoms were significantly increased among those with one or two copies of the G allele (*p* < 0.001), an effect not seen among individuals with the AA genotype (*p* = 0.07). To further examine whether differences existed between individuals with one or more copies of the G allele, orthogonal contrasts were carried out in a hierarchical linear regression. As expected, individuals with the GG and GA genotypes displayed a similar relation between childhood maltreatment and depressive symptoms, and thus did not moderate this relationship. This moderation analysis was also conducted with ethnicity as a covariate, in an effort to control for population stratification, and the analysis remained significant.

We examined the potential interactive effects between the different forms of maltreatment (i.e., psychological abuse, physical abuse, and neglect) and genotype in predicting depression. A multiple hierarchical linear regression was conducted between genotype and each of psychological abuse, physical abuse, and neglect. Genotype and maltreatment subscales were entered on the first step, and the Genotype × Maltreatment subscale interaction terms were entered on the second step. Neither psychological abuse, physical abuse, nor neglect interacted with genotype to predict depression scores, Δ *R*^2^ = 0.02, Δ *F*_(3, 272)_ = 2.07, *p* = 0.11. Thus, it seems that the relation between specific forms of maltreatment and depressive symptoms were not uniquely moderated by genotype, whereas the interaction was evident when maltreatment as a whole was considered.

It was of interest to explore potential pathways through which childhood maltreatment predicted depressive symptoms. In this regard, level of distrust was examined as a possible mediator through which childhood maltreatment predicts depression scores. As expected, distrust was positively related to depression scores, *r* = 0.53, *p* < 0.001. A mediation analyses was conducted, using bootstrapping techniques based on 5000 resamples to determine 95% confidence limits (Preacher and Hayes, 2004). The mediated effect of distrust in the relation between childhood maltreatment and severity of depressive symptoms was significant (95% CI {1.19, 2.63}), although the relation between maltreatment and depressive symptoms remained significantly evident, *b* = 1.88, *p* < 0.001. An alternative model was tested in which depressive symptoms mediated the relation between childhood maltreatment and distrust, and this model was also significant, (95% CI {0.11, 0.24}).

**Figure 2:**
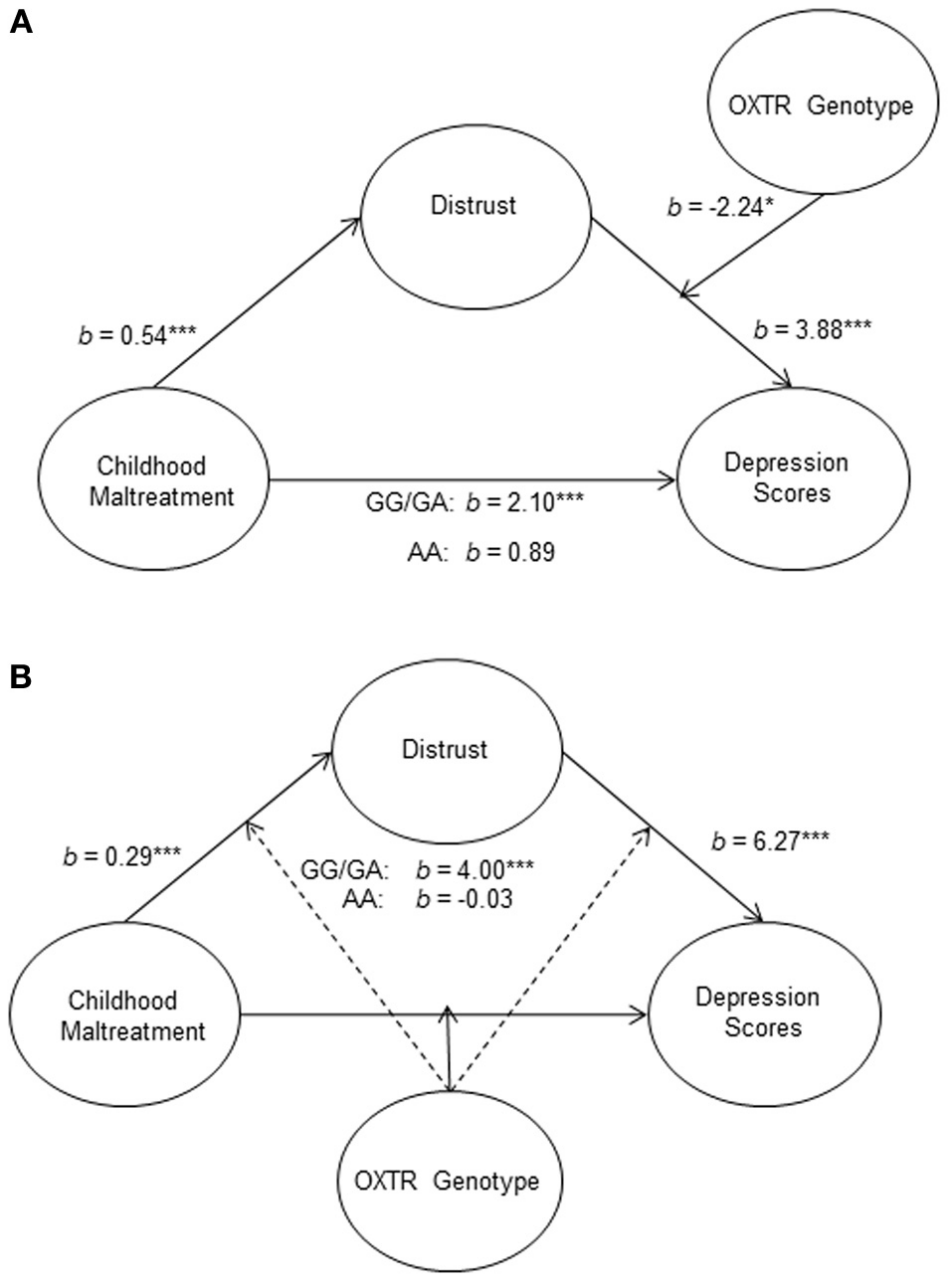
**Schematic representations of the moderated mediation models**. The relation between childhood maltreatment and depression scores through distrust was moderated by OXTR rs53576 genotype (GG/GA vs. AA). This mediation model was found only to be significant among G carriers **(A)**. An alternative model was tested, in which the relationship between childhood maltreatment and depression scores through distrust was moderated at all three pathways. This time, only the direct path was moderated and again the effect was only observed among individuals with the GG/GA genotype **(B)**. ^*^p < 0.05, and ^***^p < 0.001.

We further examined whether this mediation relationship was moderated by OXTR genotype. Moderated mediation analyses conducted using bootstrapping procedures and confidence intervals based on 5000 resamples (Preacher et al., 2007) showed that, as expected, the OXTR genotype moderated the mediating role of distrust in the relation between childhood maltreatment and depression scores (Figure 2A). Specifically, the relation between maltreatment and depression scores was mediated by distrust, but this mediated effect was only significant for G carriers. This moderated mediation analysis was also conducted with ethnicity as a covariate and remained significant.

An alternative moderation model, in which the OXTR genotype moderated the relation between childhood maltreatment and distrust was found not to be significant. However, when a final alternative model wherein the moderating effects of genotype on all three pathways were considered simultaneously (Figure 2B), only the moderation of the direct path between childhood maltreatment and depression scores accounted for unique variance. Thus, although distrust among those individuals with a G allele appeared to be more strongly associated with depressive symptoms than among those with AA genotype, when reports of childhood maltreatment were taken into consideration, their sensitivity to early-life maltreatment preclude effects attributable to distrust in the evolution of depressive symptoms.

## Discussion

In the current investigation, there was no direct associated found between the OXTR genotypes and depression scores. This is in contrast to the previous finding that A-allele carriers displayed greater depressive symptomatology (Saphire-Bernstein et al., 2011), although these variations may be due to the fact that different measures were used to assess depressive symptoms. Furthermore in the current investigation GG and GA genotypes were collapsed together, whereas Saphire-Bernstein et al. collapsed OXTR A carriers together. Importantly, in the current study, the OXTR genotype interacted with experiences of childhood maltreatment to predict depressive symptoms. Specifically, individuals with one or two copies of the G allele reported greater severity of depressive symptoms when they reported high levels of maltreatment compared to those individuals with the AA genotype. Thus, having experienced a negative early-life environment, the more sensitive G allele carriers appeared to be at greater risk of exhibiting depressive symptoms. These findings are consistent with the report that African American individuals with the GG genotype were at increased risk for emotional dysregulation provided that they experienced three or more types of childhood maltreatment (Bradley et al., 2011). Those with the GG or GA genotype in the present study also displayed higher depressive symptoms provided that they had experienced high levels childhood maltreatment. However, it is uncertain from the available data whether this relation was apparent with multiple different forms of maltreatment. This said, in the current investigation, the OXTR SNP interacted with total childhood maltreatment scores in predicting depressive symptoms and not with the individual subscales of maltreatment (i.e., psychological abuse, physical abuse, and neglect). In effect, the different forms of maltreatment do not account for unique variance, and it is all forms of maltreatment that was sufficient to increase depression symptoms among G carriers.

These findings suggest that individuals carrying one or two copies of the G allele may be more affected by previous experiences, regardless of whether it is positive or negative. Considering the research implicating the beneficial traits associated with having the G allele of the OXTR SNP, the current findings might seem counterintuitive. However, our results are in line with the view that the same genetic factors that make individuals relatively sensitive to a negative environment also influence sensitivity to a positive environment. In this respect, it has been suggested that “for better or for worse” certain genotypes are considered to promote greater plasticity and susceptible to the environment, (Belsky and Pluess, 2009; Belsky et al., 2009). Essentially, individuals with the GG or GA genotype of the OXTR SNP may thrive in a positive environment, (e.g., one that is high in social support) but, this same allele may encourage susceptibility in a negative environment. In fact, similar findings were reported with regard to the BDNF polymorphism (Val66Met) and vulnerability to childhood maltreatment. Specifically, the Val/Val genotype (which at first blush would seem to be associated with diminished vulnerability to stress-related pathology), were more negatively affected by experiences of early-life adversity compared to individuals with the Val/Met and Met/Met genotypes (Caldwell et al., 2013).

Evidently when examining the OXTR genotypes and their associations, environmental influences matter, and this extends to research focusing on other domains of the oxytocin system. In this regard, the effects of intranasal oxytocin administration on social behaviors are often moderated by contextual factors, thus leading to weak and/or inconsistent findings (Bartz et al., 2011). Furthermore, it was suggested that endogenous oxytocin levels across individuals are highly variable, and could be an indicator of sensitivity to social cues. Essentially, although high plasma oxytocin levels might be related to increased sensitivity and in turn elevated pro-social behaviors in general, the increased sensitivity among these individuals might result in elevated distress under conditions where their social needs are not met (Taylor, 2006; Bartz et al., 2011). From this perspective, oxytocin may confer a disposition toward increased sensitivity to social cues that can be either beneficial or detrimental depending on the environmental context (Bartz et al., 2011).

Beyond contextual factors, personal characteristics such as having a distrusting outlook on the world may be important when explaining the relationship between early-life adversity and depressive symptoms among specific OXTR genotypes. It has been reported that individuals with GG genotypes display greater trust in an investor-trustee money transfer game (Krueger et al., 2012). However, it has also been reported that no association existed between the OXTR SNP and human trust behaviors (Apicella et al., 2010). Thus, it was suggested that the OXTR SNP may be associated with trust-specific behaviors, but not lend itself to a general increase in trustworthy behaviors (Krueger et al., 2012). As the scale in the current investigation measured general feelings of distrust, this might account for why the OXTR genotypes did not differ in levels of distrust. Nevertheless, distrust was found to mediate the pathway between childhood maltreatment and depressive symptoms. Although, the directionality cannot be confirmed as alternative models were also significant. Furthermore, this mediation only occurred among the G carriers, and not among individuals with the AA genotype. Thus, distrust may have greater ramifications on measures of well-being among those individuals with one or more G alleles. Given that the G allele has been associated with greater levels of trust as well as greater sensitivity, it is possible that a breach of this trust could be particularly damaging for those individuals. However, when the mediation model was tested with OXTR genotype moderating all three pathways, (as seen in Figure 2B) only the direct path was significant. This suggests that, although genotype influences the extent of the relation between distrust and depressive symptoms, it seems that the differential sensitivity to negative early-life experiences is sufficiently overwhelming that when taken into consideration, the role of distrust is obfuscated. It should also be noted that when examining emotional reactions to betrayals in trust and the OXTR rs53576, no association between these variables was reported (Tabak et al., 2013). However, given the different methodologies used and especially considering the current findings were in the context of childhood maltreatment, the different outcomes are not particularly surprising.

There are several limitations associated with the current findings. Early-life maltreatment was determined based on retrospective self-reports. The use of self-reports, although common, might be biased by the individuals' current affective state, and indeed individuals might be unaware of events that occurred years earlier. The present findings are limited in the scope of the early-life adverse events that were considered, and generalizations beyond this population, in which maltreatment was moderate, would be inappropriate. Additionally, in the current investigation, G carriers were collapsed and compared to individuals with the AA genotype. This method provided the best fit to our data and has been used in previous studies, although there have also been reports in which A carriers were combined (GA/AA). However, additional analyses revealed that individuals with the GG versus GA genotypes did not differ from one another. Importantly, the functionality of the OXTR rs53576 remains unknown. Although it has been suggested that intron 3, in which this particular OXTR SNP is located, may contribute to transcription suppression (Mizumoto et al., 1997), it is also possible that the observed associations are largely due to linkage disequilibrium associations with other functional OXTR polymorphisms (Lin et al., 2007). Finally, the current study included a heterogeneous cultural sample. Ideally, a much larger sample size would allow for analyses to be done separately for each ethnic group as there may be important Gene × Environment differences across ethnicities. A larger sample size might also have permitted analyses to determine whether one or another form of abuse, interacting with genotype, was more closely aligned with depression. Thus, the inability to detect an interactive effect between the OXTR SNP and specific forms of maltreatment could be due to a lack of power stemming from the relatively small number of participants. A power analysis indicated that with the N used in the current study only a small-medium effect size could be detected.

Summarizing, the results of the present study indicated that the OXTR SNP interacts with early-life adversity in the form of maltreatment to predict depressive symptoms. Specifically, depressive symptoms were most prominent among individuals with the GG/GA genotype who experienced abuse and neglect. Being correlational, the data do not allow for causal connections to be made. Nonetheless, the present findings are consistent with the view that the OXTR genotype might favor social sensitivity so that early experiences might affect later mood states. Although the G allele has typically been characterized as being associated with beneficial attributes, it seems as if those with the G allele might be most sensitive to environmental influences, whereas individuals with the AA genotype (who are typically viewed as less socially attune or prosocial), were least affected by early-life adversity. One can imagine a breach in trust might be less detrimental to an individual who is less sensitive to their social environment, as seems to be true for the individuals with two copies of the A allele. These data provide support to the likelihood that the G allele in the OXTR SNP rs53576 is not always advantageous, and in some instances the AA genotype does not necessarily suggest an unfortunate fate.

### Conflict of interest statement

The authors declare that the research was conducted in the absence of any commercial or financial relationships that could be construed as a potential conflict of interest.
